# Higher psychological frailty index is associated with incident cardiometabolic diseases in Chinese adults aged 45 years and older

**DOI:** 10.1038/s41598-026-55226-0

**Published:** 2026-06-12

**Authors:** Qian Gui, Beijia Liu, Yue Wu, Gui Yu

**Affiliations:** 1 School of Medicine, University of Electronic Science and Technology of China, Chengdu, China; 2 Pediatric Internal Medicine, Sichuan Provincial Maternity and Child Health Care Hospital, Chengdu, China; 3 Department of Urology, Sichuan Provincial People’s Hospital, School of Medicine, University of Electronic Science and Technology of China, Chengdu, China

**Keywords:** Psychological frailty index, Cardiometabolic diseases, Cohort study, CHARLS, Cardiology, Diseases, Health care, Medical research, Risk factors

## Abstract

To prospectively investigate the association between the Psychological Frailty Index (PFI) and incident cardiometabolic diseases in middle-aged and older adults. Data were obtained from the China Health and Retirement Longitudinal Study (CHARLS). A total of 4,060 participants aged ≥ 45 years who were free of diabetes, heart disease, and stroke at baseline were included. The PFI was constructed from 15 items covering psychological distress, cognitive orientation, subjective physical vulnerability, and memory function. Incident CMD was defined as the occurrence of diabetes, heart disease, or stroke during follow-up. Multivariable Cox regression models were used to estimate hazard ratios (HRs) and 95% confidence intervals (CIs). Restricted cubic spline analyses assessed dose–response relationships, and receiver operating characteristic analyses evaluated the incremental predictive value of PFI. During a median follow-up of 24 months, higher PFI was significantly associated with increased risk of incident CMD. In the fully adjusted model, each 0.1-point increase in PFI was associated with higher risks of CMD (HR = 1.16, 95% CI: 1.09–1.23), diabetes (HR = 1.13, 95% CI: 1.02–1.26), heart disease (HR = 1.14, 95% CI: 1.05–1.24), and stroke (HR = 1.43, 95% CI: 1.24–1.64). Participants in the highest PFI group had significantly greater risks of CMD and its components than those in the lowest group, with the strongest association observed for stroke. Spline analyses suggested a progressive increase in CMD risk across higher PFI levels. Adding PFI to traditional risk factors significantly improved discrimination for overall CMD and stroke, but not for diabetes or heart disease. Higher PFI was independently associated with incident CMD, particularly stroke, among middle-aged and older adults. PFI may serve as a complementary marker for cardiometabolic risk assessment, especially for identifying individuals at elevated risk of overall CMD and stroke.

**Supplementary Information:**

The online version contains supplementary material available at 10.1038/s41598-026-55226-0.

## Introduction

With the accelerating pace of population ageing, cardiometabolic diseases—including diabetes, heart disease, and stroke—have become leading causes of disability and premature mortality among middle-aged and older adults, placing a sustained burden on healthcare systems and socioeconomic resources^1–3^. In China, the burden of these diseases is particularly significant: the 4-year cumulative incidence of heart disease is 9.51%^4^, diabetes affects 9.7% of adults^5^, and stroke has a prevalence of 3.4%^6^. These conditions collectively account for substantial morbidity, mortality, and healthcare utilization nationwide, highlighting the importance of identifying additional risk factors for these diseases. Although traditional risk factors such as obesity, hypertension, dyslipidemia, and unhealthy lifestyle behaviors are well-established, they remain insufficient to fully explain interindividual variability in disease risk^7,8^. Consequently, frailty has gained prominence as a systemic state of vulnerability, characterized by diminished multisystem physiological reserves and attenuated resilience to stressors. This concept facilitates the identification of individuals with a poorer prognosis, even when conventional risk factor profiles are comparable^9–11^. While early research primarily focused on the ‘physical frailty’ phenotype—encompassing unintentional weight loss, reduced grip strength, and decreased physical activity^12,13^—subsequent evidence has revealed that frailty is inherently multidimensional. It comprises physical, cognitive, and psychological domains, with psychological features (such as emotional distress, subjective vulnerability, and impairments in memory and attention) constituting integral yet often overlooked components of the frailty spectrum^14,15^.

Within this multidimensional framework, ‘psychological frailty’ has been proposed to describe a state of vulnerability regarding emotional regulation, cognitive processing, and subjective coping capacity^16,17^. Individual psychological indicators, such as depression, anxiety, or cognitive impairment, have been linked to atherosclerotic progression and the dysregulation of blood pressure and glycemic control^18,19^; however, these single metrics typically capture only one facet of psychological health. The Psychological Frailty Index (PFI), derived from the China Health and Retirement Longitudinal Study (CHARLS)^20^, integrates 15 items—including depressive symptoms, self-rated cognitive and memory function, and subjective physical vulnerability—within a deficit accumulation framework. This allows for the quantification of multidimensional psychological deficits as a continuous index ranging from 0 to 1, characterizing overall psychological vulnerability. Previous studies have shown that an elevated PFI is associated with diminished subjective life expectancy and increased healthcare utilization^20^. More recently, a CHARLS-based cohort study revealed that higher PFI levels were significantly associated with an increased risk of incident stroke, suggesting that psychological frailty may contribute to stroke pathogenesis via biological and behavioral pathways^21^.

Despite these advances, evidence regarding the link between psychological frailty and cardiometabolic disease remains incomplete. Firstly, most studies continue to rely on comprehensive frailty indices or physical frailty phenotypes, making it difficult to isolate the independent contribution of the psychological dimension to disease onset. Secondly, existing PFI-related investigations have primarily examined healthcare service utilization and stroke risk, leaving the risks of incident diabetes and heart disease largely unexplored. Furthermore, data enabling a horizontal comparison across the three major cardiometabolic outcomes—diabetes, heart disease, and stroke—within a single population are lacking. To address these gaps, the present study leverages the nationally representative CHARLS cohort to construct baseline PFI scores, evaluate their prospective associations with incident cardiometabolic diseases, and assess the incremental predictive value of the PFI beyond traditional risk factors.

## Methods

### Study design and data source

This study utilized data from the China Health and Retirement Longitudinal Study (CHARLS), a nationally representative, prospective cohort initiated in 2011. CHARLS employs a multistage, stratified, probability-proportional-to-size sampling strategy to recruit community-dwelling adults aged 45 years and older across China. The survey collects comprehensive information on socioeconomic status, health behaviors, chronic disease history, functional status, and psychological well-being, with follow-up assessments conducted at approximately two- to three-year intervals. Detailed descriptions of the study design, survey methodology, and response rates have been published previously^22^. The present analysis designated the fourth survey wave (July–September 2018) as baseline for exposure assessment, with the fifth wave (July–December 2020) used for outcome ascertainment. The median (IQR) follow-up duration between the two waves was 24 months. CHARLS received ethical approval from the Biomedical Ethics Review Committee of Peking University, and written informed consent was obtained from all participants. This study constitutes a secondary analysis of publicly available, de-identified data.

### Study population

**Fig. 1:**
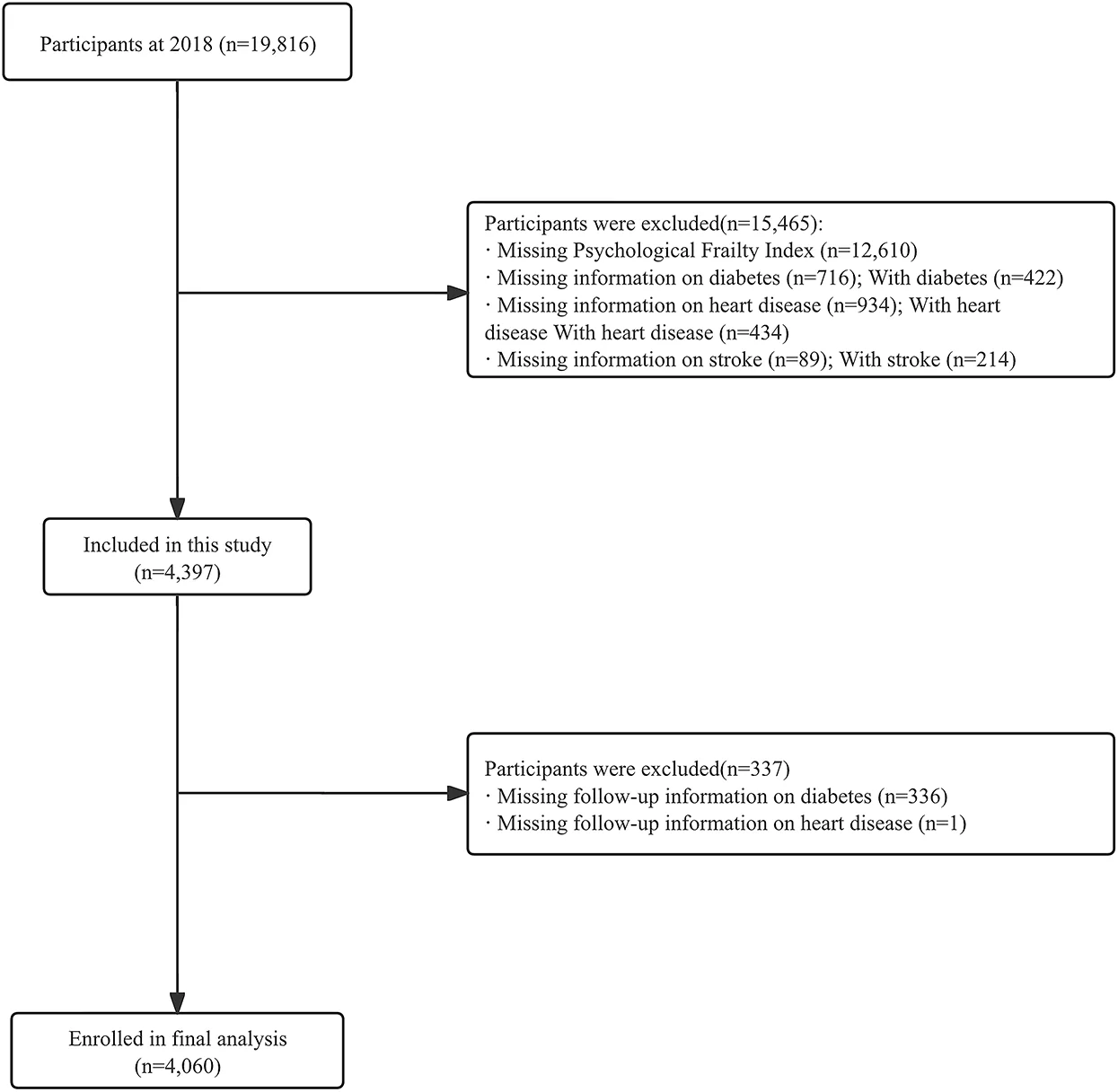
Flow chart of the study participant selection process.

Eligibility criteria were established in accordance with prior PFI development research and cardiometabolic disease cohort studies^20,23^: (1) aged ≥ 45 years at baseline; (2) completion of psychological items was defined as at least 95% of the items required for the PFI construction; (3) absence of diabetes, heart disease, or stroke at baseline; and (4) availability of follow-up information for ascertainment of incident cardiometabolic outcomes. The final analytical sample and participant selection flowchart are presented in Fig. 1.

### Assessment of the psychological frailty index

The Psychological Frailty Index (PFI) was constructed from CHARLS questionnaire data to quantify individual psychological vulnerability^20^. Guided by the deficit accumulation model, 15 items spanning emotional, cognitive, physical, and memory domains were selected, encompassing four dimensions: (1) psychological distress: depressed mood, anxiety symptoms, difficulty concentrating, feelings of helplessness/powerlessness, and loneliness; (2) cognitive orientation: errors in identifying current date, year, season, and residential district; (3) physical vulnerability: subjective decline in strength, physical exhaustion, restricted physical activity, and limitations in daily functioning; and (4) memory function: self-reported memory impairment and forgetfulness. Item coding and scoring procedures are detailed in Supplementary Table 1. Although conventional frailty indices often include a larger number of deficits to improve measurement stability^24^, the present PFI was adopted from a previously developed CHARLS-based psychological frailty framework and was designed to capture domain-specific psychological vulnerability rather than overall biological frailty. The PFI total score was calculated by summing all 15 items and dividing by the total number of items, yielding a continuous index ranging from 0 to 1.

### Ascertainment of cardiometabolic diseases

The primary endpoint of this study was cardiometabolic diseases (CMD), defined as the occurrence of any diabetes, stroke, or heart disease during follow-up. Outcome data were derived from the CHARLS follow-up questionnaire module enquiring whether participants had received a physician diagnosis of specific conditions, supplemented by related questions. Among participants free of diabetes at baseline, incident diabetes was defined as first self-report of a physician diagnosis of diabetes or current use of glucose-lowering medications during follow-up. Among those without baseline heart disease, incident heart disease was defined as first self-report of a physician diagnosis of myocardial infarction, heart disease, or heart failure^25^. Among participants with no history of stroke at baseline, incident stroke was defined as first self-report of a physician diagnosis of stroke^26^.

### Covariate collection and definitions

Covariates were selected based on established literature and their potential associations with both psychological frailty and cardiometabolic diseases^23^. All covariates were measured at baseline and were used only as adjustment variables in the multivariable models. Demographic covariates included age, sex, residence, educational attainment, and marital status. Age was treated as a continuous variable; residence was classified as urban or rural; educational attainment was categorized as illiterate or incomplete primary school, primary or secondary school, and high school or above; and marital status was classified as married versus other status. Lifestyle covariates included current smoking status and alcohol consumption within the preceding year, both coded as yes or no. Chronic disease-related covariates included hypertension, dyslipidemia, lung disease, liver disease, and kidney disease, which were entered into the models as individual baseline comorbidities. The outcome variables were incident CMD, diabetes, heart disease, and stroke during follow-up. All covariates were ascertained at baseline to preserve the temporal sequence between exposure and outcome.

### Statistical analysis

Baseline characteristics were summarized according to PFI-defined tertiles groups (T1, T2, T3). Categorical variables were expressed as frequencies (percentages), whilst continuous variables were presented as means ± standard deviations or medians (interquartile ranges), as appropriate. Between-group comparisons were performed using chi-squared tests for categorical variables and t-tests or Wilcoxon rank-sum tests for continuous variables. Multivariable Cox regression models were constructed with incident CMD, diabetes, heart disease, and stroke as dependent variables and PFI as the primary independent variable, yielding hazard ratio (HR) with corresponding 95% confidence intervals. For continuous analyses, PFI was rescaled so that HRs corresponded to each 0.1-point increase. A sequential adjustment strategy was employed: Model 1 represented the unadjusted crude model; Model 2 adjusted for age and sex; Model 3 additionally adjusted for educational attainment, residential location, marital status, smoking, and alcohol consumption; Model 4 further incorporated chronic disease history (hypertension, dyslipidemia, pulmonary disease, hepatic disease, and renal disease) to evaluate the independent effect of psychological frailty on each outcome. PFI was modeled both as a continuous variable and as a categorical variable based on tertiles groups (T1, T2, T3) to assess linear trends and risk differences across exposure strata. To explore potential non-linear associations between PFI and each outcome, restricted cubic splines were incorporated into Cox regression models, enabling visualization of risk trajectories across the PFI continuum. Subgroup analyses were conducted stratified by age, sex, residential location, marital status, alcohol consumption status, and presence of hypertension. To test for potential effect modification, multiplicative interaction terms between PFI and each subgroup variable were added one at a time to the fully adjusted model (Model 4), which also included all covariates. All statistical analyses were performed using R software (version 4.2.2) and the Free Statistics analytical platform (version 2.3). Statistical significance was defined as a two-sided *P* value < 0.05.

## Results

### Baseline characteristics

A total of 4,060 participants were included and divided into three groups according to tertiles of PFI: T1 (*n* = 1,139), T2 (*n* = 1,553), and T3 (*n* = 1,368) (Table 1). Mean age increased progressively across PFI groups (66.8 ± 5.4, 67.5 ± 6.0, and 68.5 ± 6.4 years, respectively; *P* < 0.001). Participants in the highest PFI group were more likely to be female, rural residents, less educated, unmarried, non-smokers, and non-drinkers (all *P* < 0.001). The prevalence of hypertension and kidney disease was also higher in T3 than in T1 and T2 (*P* = 0.034 and *P* = 0.001, respectively), whereas dyslipidemia, lung disease, and liver disease showed no significant differences across groups. In addition, the cumulative incidence of diabetes, heart disease, stroke, and CMD during follow-up increased across PFI tertiles, with the highest rates observed in T3 (5.3%, 8.5%, 3.4%, and 15.5%, respectively; all *P* < 0.01).

**Table 1:** Baseline characteristics by PFI status groups.

Variables	Total (*n* = 4060)	T1 (*n* = 1139)	T2 (*n* = 1553)	T3 (*n* = 1368)	*P*
Age, Mean ± SD	67.6 ± 6.0	66.8 ± 5.4	67.5 ± 6.0	68.5 ± 6.4	< 0.001
Gender, n (%)					< 0.001
Female	1786 (44.0)	332 (29.1)	605 (39)	849 (62.1)	
Male	2274 (56.0)	807 (70.9)	948 (61)	519 (37.9)	
Residence ^a^, n (%)					< 0.001
Rural	3199 (78.8)	764 (67.1)	1220 (78.6)	1215 (88.8)	
Urban	861 (21.2)	375 (32.9)	333 (21.4)	153 (11.2)	
Education, n (%)					< 0.001
Below primary school	1932 (47.6)	314 (27.6)	676 (43.5)	942 (68.9)	
Primary school	984 (24.2)	308 (27)	409 (26.3)	267 (19.5)	
Middle school	750 (18.5)	318 (27.9)	314 (20.2)	118 (8.6)	
High school and above	394 ( 9.7)	199 (17.5)	154 (9.9)	41 (3)	
Marital status, n (%)					< 0.001
Married	3283 (80.9)	968 (85)	1277 (82.2)	1038 (75.9)	
Other	777 (19.1)	171 (15)	276 (17.8)	330 (24.1)	
Smoke ^a^, n (%)					< 0.001
No	3645 (89.8)	997 (87.5)	1387 (89.3)	1261 (92.2)	
Yes	415 (10.2)	142 (12.5)	166 (10.7)	107 (7.8)	
Drink, n (%)					< 0.001
No	2501 (61.6)	582 (51.1)	929 (59.8)	990 (72.4)	
Yes	1559 (38.4)	557 (48.9)	624 (40.2)	378 (27.6)	
Hypertension ^a^, n (%)					0.034
No	3576 (88.1)	1025 (90)	1366 (88)	1185 (86.6)	
Yes	484 (11.9)	114 (10)	187 (12)	183 (13.4)	
Dyslipidemia ^a^, n (%)					0.479
No	3768 (92.8)	1053 (92.4)	1451 (93.4)	1264 (92.4)	
Yes	292 ( 7.2)	86 (7.6)	102 (6.6)	104 (7.6)	
Lung disease ^a^, n (%)					0.058
No	3875 (95.4)	1096 (96.2)	1488 (95.8)	1291 (94.4)	
Yes	185 ( 4.6)	43 (3.8)	65 (4.2)	77 (5.6)	
Liver disease ^a^, n (%)					0.758
No	3961 (97.6)	1114 (97.8)	1512 (97.4)	1335 (97.6)	
Yes	99 ( 2.4)	25 (2.2)	41 (2.6)	33 (2.4)	
Kidney disease ^a^, n (%)					0.001
No	3940 (97.0)	1118 (98.2)	1512 (97.4)	1310 (95.8)	
Yes	120 ( 3.0)	21 (1.8)	41 (2.6)	58 (4.2)	
Diabetes, n (%)					0.006
No	3901 (96.1)	1100 (96.6)	1505 (96.9)	1296 (94.7)	
Yes	159 ( 3.9)	39 (3.4)	48 (3.1)	72 (5.3)	
Heart disease, n (%)					< 0.001
No	3795 (93.5)	1087 (95.4)	1456 (93.8)	1252 (91.5)	
Yes	265 ( 6.5)	52 (4.6)	97 (6.2)	116 (8.5)	
Stroke, n (%)					< 0.001
No	3976 (97.9)	1127 (98.9)	1527 (98.3)	1322 (96.6)	
Yes	84 ( 2.1)	12 (1.1)	26 (1.7)	46 (3.4)	
CMD, n (%)					< 0.001
No	3582 (88.2)	1038 (91.1)	1388 (89.4)	1156 (84.5)	
Yes	478 (11.8)	101 (8.9)	165 (10.6)	212 (15.5)	
PFI, Mean ± SD	0.3 ± 0.2	0.1 ± 0.0	0.3 ± 0.0	0.5 ± 0.1	< 0.001

Abbreviations: SD, standard deviation; CMD, cardiometabolic diseases; T, tertiles.

a Missing data: 46 for age; 24 for residence; 818 for smoke; 972 for hypertension; 315 for dyslipidemia; 384 for lung diseases; 126 for liver diseases; 209 for kidney diseases.

### Association between PFI and cardiometabolic diseases

Multivariable Cox regression analyses revealed significant positive associations between PFI and incident cardiometabolic diseases following sequential adjustment for potential confounders (Table 2). In the fully adjusted model (Model 4), each 0.1-point increase in PFI was associated with a 16% increased risk of CMD (HR = 1.16, 95% CI: 1.09–1.23), a 13% increased risk of diabetes (HR = 1.13, 95% CI: 1.02–1.26), a 14% increased risk of heart disease (HR = 1.14, 95% CI: 1.05–1.24), and a 43% greater risk of stroke (HR = 1.43, 95% CI: 1.24–1.64).

Categorical analyses further supported a graded association between PFI levels and incident cardiometabolic outcomes. Compared with participants in the lowest PFI group (T1), those in the highest PFI group (T3) had significantly higher risks of overall CMD (HR = 1.84; 95% CI: 1.42–2.39; *P* < 0.001), diabetes (HR = 1.56; 95% CI: 1.01–2.41; *P* = 0.044), heart disease (HR = 1.90; 95% CI: 1.33–2.71; *P* < 0.001), and stroke (HR = 3.24; 95% CI: 1.66–6.33; *P* = 0.001) in the fully adjusted model. The intermediate PFI group (T2) showed a significantly increased risk only for heart disease (HR = 1.42; 95% CI: 1.01–2.00; *P* = 0.043), whereas associations with overall CMD, diabetes, and stroke did not reach statistical significance.

**Table 2:** Association between PFI and cardiometabolic outcomes.

Variable	Model 1		Model 2		Model 3		Model 4	
	HR (95%CI)	*P* value	HR (95%CI)	*P* value	HR (95%CI)	*P* value	HR (95%CI)	*P* value
**CMD**								
PFI	1.16 (1.11 ~ 1.23)	< 0.001	1.16 (1.1 ~ 1.22)	< 0.001	1.18 (1.11 ~ 1.25)	< 0.001	1.16 (1.09 ~ 1.23)	< 0.001
PFI group								
T1	1(Ref)		1(Ref)		1(Ref)		1(Ref)	
T2	1.23 (0.96 ~ 1.58)	0.097	1.22 (0.95 ~ 1.57)	0.112	1.24 (0.97 ~ 1.6)	0.089	1.24 (0.97 ~ 1.6)	0.089
T3	1.86 (1.47 ~ 2.36)	< 0.001	1.82 (1.42 ~ 2.32)	< 0.001	1.89 (1.46 ~ 2.45)	< 0.001	1.84 (1.42 ~ 2.39)	< 0.001
Trend test	1.38 (1.23 ~ 1.56)	< 0.001	1.36 (1.21 ~ 1.54)	< 0.001	1.39 (1.22 ~ 1.58)	< 0.001	1.37 (1.2 ~ 1.56)	< 0.001
**Diabetes**								
PFI	1.15 (1.05 ~ 1.26)	0.002	1.14 (1.03 ~ 1.25)	0.009	1.16 (1.04 ~ 1.28)	0.006	1.13 (1.02 ~ 1.26)	0.018
PFI group								
T1	1(Ref)		1(Ref)		1(Ref)		1(Ref)	
T2	0.92 (0.6 ~ 1.4)	0.686	0.91 (0.59 ~ 1.39)	0.658	0.93 (0.61 ~ 1.44)	0.757	0.93 (0.6 ~ 1.42)	0.725
T3	1.61 (1.09 ~ 2.38)	0.017	1.54 (1.03 ~ 2.31)	0.037	1.63 (1.06 ~ 2.51)	0.026	1.56 (1.01 ~ 2.41)	0.044
Trend test	1.32 (1.07 ~ 1.61)	0.008	1.28 (1.04 ~ 1.58)	0.022	1.32 (1.05 ~ 1.65)	0.016	1.29 (1.03 ~ 1.61)	0.028
**Heart disease**								
PFI	1.15 (1.08 ~ 1.24)	< 0.001	1.12 (1.04 ~ 1.21)	0.002	1.15 (1.06 ~ 1.25)	0.001	1.14 (1.05 ~ 1.24)	0.002
PFI group								
T1	1(Ref)		1(Ref)		1(Ref)		1(Ref)	
T2	1.41 (1.01 ~ 1.97)	0.046	1.37 (0.97 ~ 1.91)	0.071	1.41 (1 ~ 1.99)	0.048	1.42 (1.01 ~ 2)	0.043
T3	1.96 (1.41 ~ 2.71)	< 0.001	1.78 (1.27 ~ 2.5)	0.001	1.94 (1.36 ~ 2.76)	< 0.001	1.9 (1.33 ~ 2.71)	< 0.001
Trend test	1.4 (1.19 ~ 1.64)	< 0.001	1.33 (1.13 ~ 1.57)	0.001	1.39 (1.17 ~ 1.65)	< 0.001	1.37 (1.15 ~ 1.63)	< 0.001
**Stroke**								
PFI	1.36 (1.21 ~ 1.53)	< 0.001	1.47 (1.3 ~ 1.67)	< 0.001	1.43 (1.25 ~ 1.64)	< 0.001	1.43 (1.24 ~ 1.64)	< 0.001
PFI group								
T1	1(Ref)		1(Ref)		1(Ref)		1(Ref)	
T2	1.63 (0.82 ~ 3.22)	0.164	1.7 (0.86 ~ 3.38)	0.127	1.53 (0.77 ~ 3.05)	0.225	1.54 (0.77 ~ 3.08)	0.217
T3	3.33 (1.76 ~ 6.28)	< 0.001	3.9 (2.04 ~ 7.48)	< 0.001	3.23 (1.65 ~ 6.3)	0.001	3.24 (1.66 ~ 6.33)	0.001
Trend test	1.88 (1.39 ~ 2.54)	< 0.001	2.05 (1.5 ~ 2.8)	< 0.001	1.87 (1.35 ~ 2.58)	< 0.001	1.87 (1.35 ~ 2.58)	< 0.001

Abbreviations: T, tertiles; HR, hazard ratio; Ref, reference.

### Restricted cubic spline analysis

**Fig. 2:**
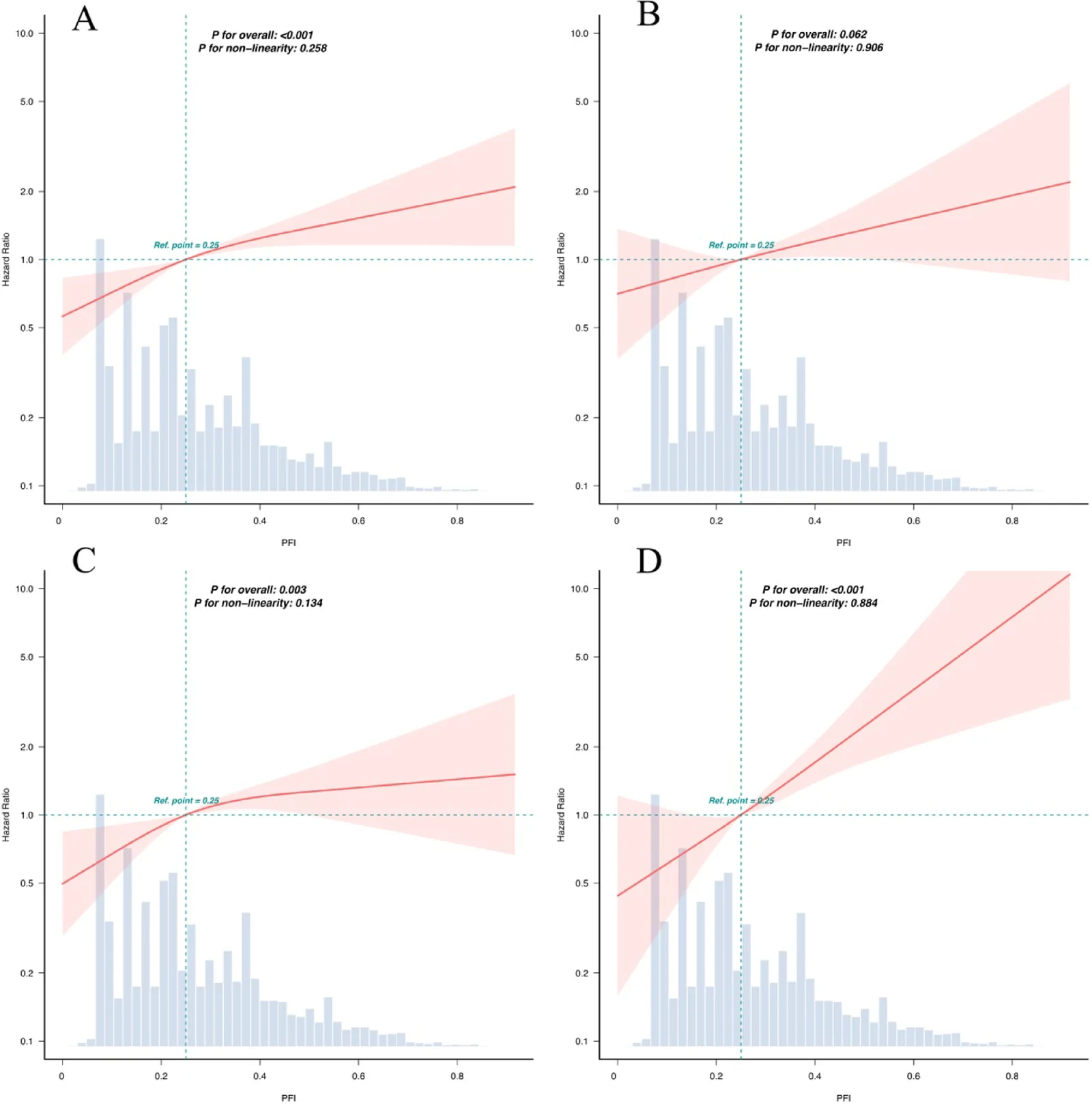
Dose–response associations between the PFI and incident cardiometabolic diseases. Panel **A**: overall cardiometabolic diseases (CMD), Panel **B**: diabetes, Panel **C**: heart disease, Panel **D**: stroke.

Restricted cubic spline analyses were used to examine the dose–response relationships between PFI and incident cardiometabolic diseases (Fig. 2). Higher PFI was significantly associated with increased risks of overall cardiometabolic diseases (*P* for overall < 0.001), heart disease (*P* for overall = 0.003), and stroke (*P* for overall < 0.001). The overall association for diabetes was marginal and did not reach statistical significance in the spline model, although the continuous Cox model showed a significant positive association. Tests for non-linearity were non-significant for all outcomes, including overall cardiometabolic diseases, diabetes, heart disease, and stroke (all P for non-linearity > 0.05), suggesting that the observed associations were primarily linear. Therefore, elevated PFI was associated with a progressively higher risk of cardiometabolic diseases, particularly heart disease and stroke, without evidence of a significant non-linear dose–response pattern.

### Receiver operating characteristic analysis

**Fig. 3:**
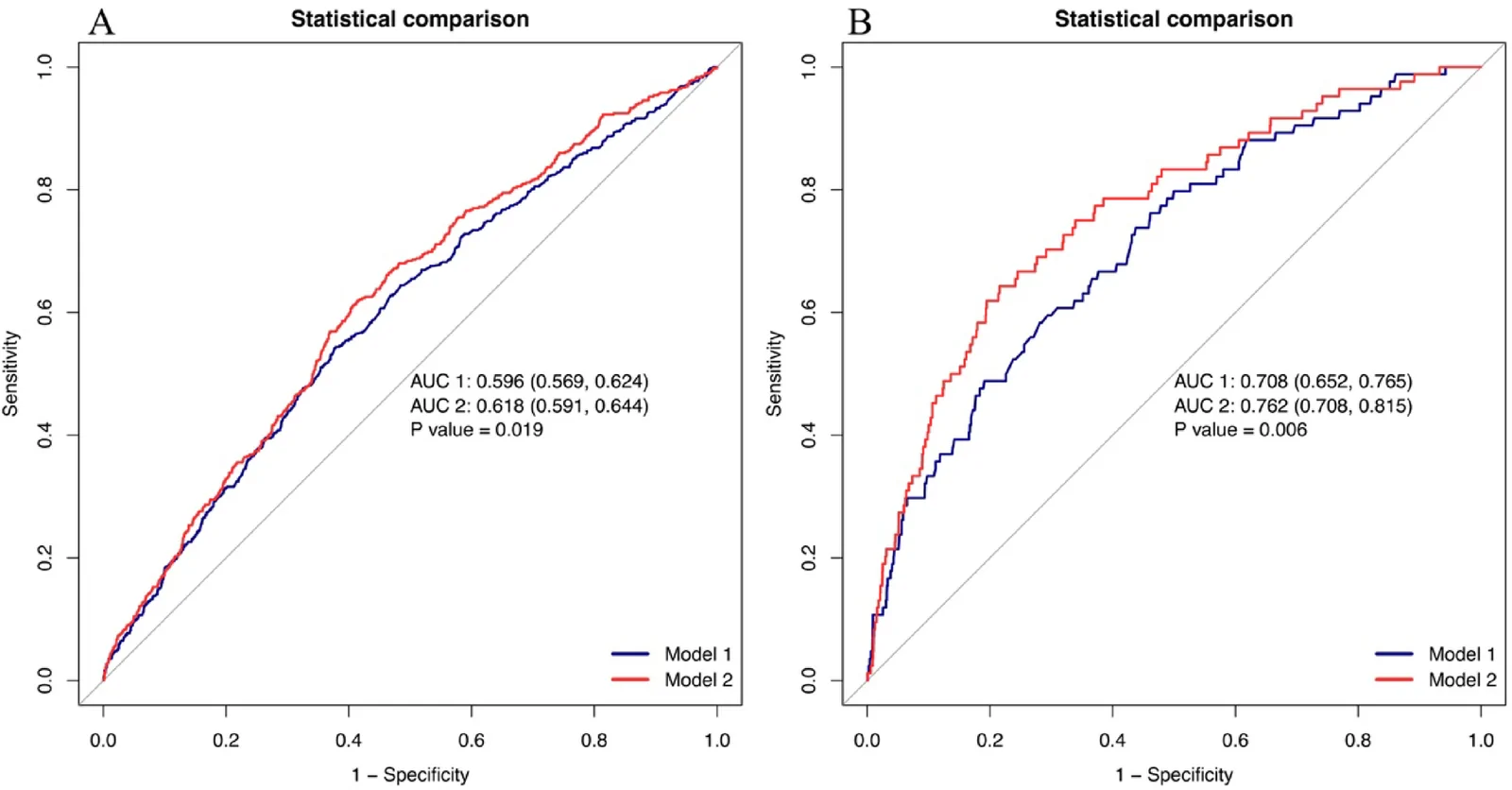
Comparison of AUC between Model 1 (traditional risk factors) and Model 2 (traditional risk factors + PFI) for incident cardiometabolic diseases and stroke.

To further evaluate whether the PFI provided incremental predictive information beyond traditional risk factors, we compared the discriminative performance of two nested models. Model 1 included demographic characteristics and traditional risk factors, including age, gender, residence, education, marital status, smoking, drinking, hypertension, dyslipidemia, lung disease, liver disease, and kidney disease. Model 2 included all variables in Model 1 plus the PFI. The inclusion of the PFI significantly improved model discrimination for overall cardiometabolic diseases, with the AUC increasing from 0.596 to 0.618 (*P* = 0.019), and for stroke, with the AUC increasing from 0.708 to 0.762 (*P* = 0.006) (Fig. 3). However, adding the PFI did not significantly improve the predictive performance for diabetes or heart disease. These results indicate that the PFI has incremental predictive value for overall cardiometabolic diseases and stroke beyond traditional risk factors.

### Subgroup analyses

**Fig. 4:**
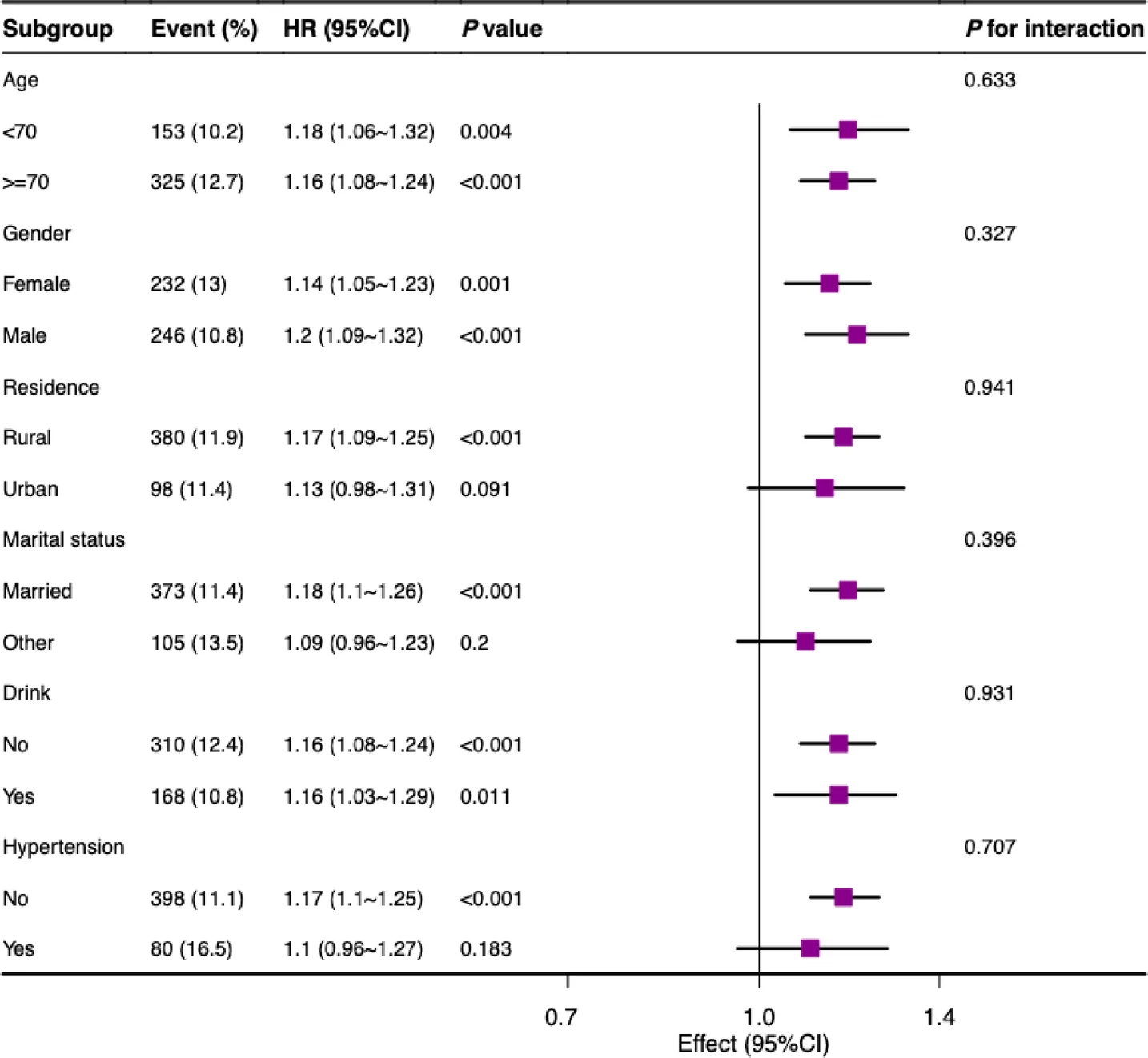
Subgroup analysis of the association between CMD and PFI.

Subgroup analyses further supported the robustness of the association between the PFI and incident cardiometabolic diseases (Figure 4). Higher PFI was associated with increased cardiometabolic disease risk across most predefined subgroups, including strata defined by age, gender, residence, marital status, drinking status, and hypertension status. The interaction tests were not statistically significant for any subgroup variable, suggesting no evidence of effect modification by these baseline characteristics.

## Discussion

In this large-scale, nationally representative cohort study, we examined the association between the Psychological Frailty Index (PFI) and incident cardiometabolic diseases (CMD), including diabetes, heart disease, and stroke, using data from the China Health and Retirement Longitudinal Study (CHARLS). Our primary finding is that higher PFI scores are significantly associated with an increased risk of overall CMD, with a linear dose-response relationship. Specifically, each 0.1-point increment in PFI was linked to a 16% higher risk of CMD. Notably, the association with stroke was the strongest, with individuals in the highest PFI group showing more than three times the risk compared to those in the lowest group. While PFI was also associated with increased risks of diabetes and heart disease, these associations were somewhat weaker than for CMD and stroke. Restricted cubic spline analyses confirmed the linear nature of these associations, indicating that even modest psychological frailty increases the risk of adverse metabolic and vascular outcomes. Additionally, ROC analysis demonstrated that adding PFI to traditional risk factors significantly improved model discrimination for CMD and stroke but not for diabetes or heart disease.

These findings corroborate and extend prior evidence. A systematic review by Miao et al.^27^ established significant associations between comprehensive frailty indices and cardiovascular events, whilst a meta-analysis by Gao et al.^28^ revealed bidirectional relationships between frailty and type 2 diabetes. However, these investigations predominantly employed physical frailty phenotypes or composite frailty indices, precluding delineation of the independent contribution of psychological dimensions. More recently, Wu et al.^29^ demonstrated independent associations between depressive symptoms and cardiovascular risk using data from the English Longitudinal Study of Ageing, yet their analysis focused solely on a single psychological indicator. By employing a multidimensional PFI integrating depressive symptoms, cognitive function, and subjective vulnerability, the present study validates the predictive value of cumulative psychological deficits for cardiometabolic outcomes. The PFI–stroke association reported by Guo et al.^21^ using CHARLS data aligns with our findings, whilst our study extends this evidence base to encompass diabetes and heart disease, thereby addressing a gap in the existing literature.

The mechanisms through which psychological frailty influences cardiometabolic diseases may be understood from multiple perspectives. First, depression and anxiety activate the hypothalamic-pituitary-adrenal axis, resulting in sustained cortisol elevation that subsequently promotes insulin resistance and endothelial dysfunction^30,31^. Second, psychologically frail individuals frequently exhibit chronic low-grade inflammation; elevated levels of inflammatory mediators such as C-reactive protein and interleukin-6 accelerate atherosclerotic progression^32,33^. The recently proposed ‘inflammaging’ theory provides a valuable conceptual framework for understanding the nexus between psychological frailty and vascular pathology^34,35^. Additionally, cognitive decline may compromise self-management capabilities, leading to diminished treatment adherence and suboptimal risk factor control^36^. The notably stronger association between PFI and stroke compared with diabetes and heart disease merits particular attention and may reflect the heightened susceptibility of cerebrovascular structures to psychological stress. Evidence indicates that chronic psychological stress preferentially damages the cerebrovascular system through mechanisms including sympathetic hyperactivation, increased blood pressure variability, and carotid intima-media thickening^37,38^.

This study has several strengths, including the use of a large-scale, nationally representative cohort (CHARLS) and a prospective design that minimizes reverse causation bias. However, there are limitations that should be acknowledged. The outcome assessment relied on self-reported diagnoses, which could introduce recall bias or underreporting. The relatively short follow-up duration (median 24 months) may limit the ability to capture long-term effects of psychological frailty on cardiometabolic disease development. Additionally, because the PFI was assessed only at baseline, we were unable to assess how changes in psychological frailty over time might influence disease risk. Fourth, because the PFI included only 15 items, its measurement stability may be more limited than that of conventional frailty indices with larger deficit pools. Future studies should further validate and refine psychological frailty measures in independent cohorts.

## Conclusion

In conclusion, our study highlights the Psychological Frailty Index (PFI) as an important independent predictor of incident cardiometabolic diseases, particularly for overall cardiometabolic diseases (CMD) and stroke. Our results show that higher PFI scores are significantly associated with increased risks of these outcomes, with the strongest predictive value observed for stroke. Importantly, the inclusion of PFI in models with traditional risk factors enhanced the ability to discriminate risk for CMD and stroke, underscoring its incremental value in predicting cardiometabolic disease risk beyond conventional measures. These findings suggest that early identification of psychological frailty may serve as a valuable tool for targeted prevention strategies aimed at reducing the burden of cardiometabolic diseases in aging populations.

## Supplementary Information

Below is the link to the electronic supplementary material.

Supplementary Material 1

## Data Availability

The datasets analyzed during this study are publicly available from the China Health and Retirement Longitudinal Study (CHARLS) repository at http://charls.pku.edu.cn/en.

## Abbreviations

PFI: Psychological Frailty Index
CHARLS: China Health and Retirement Longitudinal Study
HR: Hazard Ratio
CI: Confidence Interval
RCS: Restricted Cubic Splines
AUC: Area Under the Curve
