# Supplementary material for: Higher psychological frailty index is associated with incident cardiometabolic diseases in Chinese adults aged 45 years and older
Source: Sci Rep. 2026 Jun 12;16:26786. doi: 10.1038/s41598-026-55226-0 (PMC13507105; doi:10.1038/s41598-026-55226-0)
Supplement: Supplementary file 1 [file 41598_2026_55226_MOESM1_ESM.docx]

**Supplementary material**

**Supplementary Table 1** Variable List for the Psychological Frailty Index

| **Item** | **Variable** | **Definition** | **Categories** | **Coding in PFI item** |
| --- | --- | --- | --- | --- |
| 1 | DC011 | Depression: I felt depressed | rarely or none of the time (<1 day), some or a little of the time (1-2 days), occasionally or a moderate amount of the time (3-4 days), most or all of the time (5-7 days) | 0, 0.5, 0.75, 1, respectively |
| 2 | DC001_W4 | Disorientation: checking year | correct, error | Categorized 0/1 |
| 3 | DC002_W4 | Disorientation: checking season | correct, error | Categorized 0/1 |
| 4 | DC007_W4 | Disorientation: checking the state | correct, error | Categorized 0/1 |
| 5 | DC008_W4 | Disorientation: checking county | correct, error | Categorized 0/1 |
| 6 | DC010 | Attentional problem: I had trouble keeping my mind on what I was doing | rarely or none of the time (<1 day), some or a little of the time (1-2 days), occasionally or a moderate amount of the time (3-4 days), most or all of the time (5-7 days) | 0, 0.5, 0.75, 1, respectively |
| 7 | DD012_W4 | Memory impairment: recognize faces of families and friends | much better, improved a little, not much changed, a little worse, much worse | 0, 0.25, 0.5, 0.75, 1, respectively |
| 8 | DC009 | Anxiety: bothered by things | rarely or none of the time (<1 day), some or a little of the time (1-2 days), occasionally or a moderate amount of the time (3-4 days), most or all of the time (5-7 days) | 0, 0.5, 0.75, 1, respectively |
| 9 | DC017 | Loneliness: I felt lonely | rarely or none of the time (<1 day), some or a little of the time (1-2 days), occasionally or a moderate amount of the time (3-4 days), most or all of the time (5-7 days) | 0, 0.5, 0.75, 1, respectively |
| 10 | DC012 | Powerlessness: I felt everything I did was an effort | rarely or none of the time (<1 day), some or a little of the time (1-2 days), occasionally or a moderate amount of the time (3-4 days), most or all of the time (5-7 days) | 0, 0.5, 0.75, 1, respectively |
| 11 | DB001 | limited physical activity: difficulty with running or jogging about 1km | do not have any difficulty, have difficulty but can still do it, have difficulty and need help, cannot do it | 0, 0.5, 0.75, 1, respectively |
| 12 | DB004 | Physical exhaustion: difficulty with getting up from a chair | do not have any difficulty, have difficulty but can still do it, have difficulty and need help, cannot do it | 0, 0.5, 0.75, 1, respectively |
| 13 | DB007 | Functional limitations (limitations in stretching) | do not have any difficulty, have difficulty but can still do it, have difficulty and need help, cannot do it | 0, 0.5, 0.75, 1, respectively |
| 14 | DB008 | Decreased strength | do not have any difficulty, have difficulty but can still do it, have difficulty and need help, cannot do it | 0, 0.5, 0.75, 1, respectively |
| 15 | DD013_W4 | Memory loss: remember names of families and friends | much better, improved a little, not much changed, a little worse, much worse | 0, 0.25, 0.5, 0.75, 1, respectively |
